# Perfluoroalkane Acids: Apelberg et al. Respond

**DOI:** 10.1289/ehp.11036R

**Published:** 2008-06

**Authors:** Benjamin J. Apelberg, Lynn R. Goldman, Rolf U. Halden, Frank R. Witter, Julie B. Herbstman, Larry L. Needham

**Affiliations:** Johns Hopkins Bloomberg School of Public Health, Baltimore, Maryland, E-mail: Lgoldman@jhsph.edu; Johns Hopkins University, School of Medicine, Baltimore, Maryland; Columbia University Mailman School of Public Health, New York, New York; Centers for Disease Control and Prevention, Atlanta, Georgia

We thank Scialli for his interest in our study (Apelberg et al. 2007). As he notes, we recognize that several factors could be responsible for the relationships observed between cord serum concentrations of perfluorooctane sulfonate/perfluorooctanoate (PFOS/PFOA) and birth weight, head circumference, and ponderal index in our study. Although diet may be a source of exposure (including consumption of polyfluoroalkyl compounds used in fast-food packaging), we are not aware of any evidence that such diets are associated with smaller size at birth. In fact, they may be related to obesity, which is associated with larger birth size (Surkan et al. 2004). Despite existing knowledge gaps on exposure pathways and the role of dietary intake, we do know that in our study, adjusting for body mass index of the mother had little impact on the associations observed.

Scialli posits that there may be a role of reduced plasma or body water volume on the associations observed. As we described in our article (Apelberg et al. 2007), both preeclampsia and pregnancy-induced hypertension (PIH) are associated with poor maternal plasma volume expansion (Salas et al. 2006), as is placental weight (Salas et al. 1993). However, cord concentrations of PFOS and PFOA were not elevated among mothers with preeclampsia or PIH, and adjustment for these conditions did not appreciably alter the observed associations. Likewise, adjustment for placental weight, which may be associated with plasma volume of the infant, did not alter these associations. Despite theoretical considerations, we have not found support for this hypothesis. Further research is needed to better understand the pathways of human exposure and the role that pharmacokinetics of these compounds in the human body may play in the observed associations.

## References

[b1-ehp0116-a0238b] Apelberg BJ, Witter FR, Herbstman JB, Calafat AM, Halden RU, Needham LL (2007). Cord serum concentrations of perfluorooctane sulfonate (PFOS) and perfluorooctanoate (PFOA) in relation to weight and size at birth. Environ Health Perspect.

[b2-ehp0116-a0238b] Salas SP, Marshall G, Gutierrez BL, Rosso P (2006). Time course of maternal plasma volume and hormonal changes in women with preeclampsia or fetal growth restriction. Hypertension.

[b3-ehp0116-a0238b] Salas SP, Rosso P, Espinoza R, Robert JA, Valdes G, Donoso E (1993). Maternal plasma volume expansion and hormonal changes in women with idiopathic fetal growth retardation. Obstet Gynecol.

[b4-ehp0116-a0238b] Surkan PJ, Hsieh CC, Johansson AL, Dickman PW, Cnattingius S (2004). Reasons for increasing trends in large for gestational age births. Obstet Gynecol.

